# Role of microglial amylin receptors in mediating beta amyloid (Aβ)-induced inflammation

**DOI:** 10.1186/s12974-017-0972-9

**Published:** 2017-10-06

**Authors:** Wen Fu, Vlatka Vukojevic, Aarti Patel, Rania Soudy, David MacTavish, David Westaway, Kamaljit Kaur, Valeri Goncharuk, Jack Jhamandas

**Affiliations:** 1 Department of Medicine (Neurology), Neuroscience and Mental Health Institute, University of Alberta, 530 Heritage Medical Research Centre, Edmonton, AB T6G 2S2 Canada; 2 Faculty of Pharmacy, Cairo University, Cairo, Egypt; 3 Department of Biochemistry, University of Alberta, Edmonton, AB Canada; 4 Centre for Prions and Protein Folding Diseases, University of Alberta, Edmonton, AB Canada; 5 Chapman University School of Pharmacy, Irvine, CA USA; 6 Russian Cardiology Research Center, Moscow, Russia

**Keywords:** Microglia, Amylin receptor, β-amyloid, AC253 peptide, Inflammasome, NLRP3, Caspase-1, Cytokines, Alzheimer’s disease

## Abstract

**Background:**

Neuroinflammation in the brain consequent to activation of microglia is viewed as an important component of Alzheimer’s disease (AD) pathology. Amyloid beta (Aβ) protein is known to activate microglia and unleash an inflammatory cascade that eventually results in neuronal dysfunction and death. In this study, we sought to identify the presence of amylin receptors on human fetal and murine microglia and determine whether Aβ activation of the inflammasome complex and subsequent release of cytokines is mediated through these receptors.

**Methods:**

The presence of dimeric components of the amylin receptor (calcitonin receptor and receptor activity modifying protein 3) were first immunohistochemically identified on microglia. Purified human fetal microglial (HFM) cultures were incubated with an in vivo microglial marker, DyLight 594-conjugated tomato lectin, and loaded with the membrane-permeant green fluorescent dye, Fluo-8L-AM for measurements of intracellular calcium [Ca^2+^]i. HFM and BV-2 cells were primed with lipopolysaccharide and then exposed to either human amylin or soluble oligomeric Aβ_1–42_ prior to treatment with and without the amylin receptor antagonist, AC253. Changes in the inflammasome complex, NLRP3 and caspase-1, were examined in treated cell cultures with Western blot and fluorometric assays. RT-PCR measurements were performed to assess cytokine release. Finally, in vivo studies were performed in transgenic mouse model of AD (5xFAD) to examine the effects of systemic administration of AC253 on markers of neuroinflammation in the brain.

**Results:**

Acute applications of human amylin or Aβ_1–42_ resulted in an increase in [Ca^2+^]i that could be blocked by the amylin receptor antagonist, AC253. Activation of the NLRP3 and caspase-1 and subsequent release of cytokines, TNFα and IL-1β, was diminished by AC253 pretreatment of HFMs and BV2 cells. In vivo, intraperitoneal administration of AC253 resulted in a reduction in microglial markers (Iba-1 and CD68), caspase-1, TNFα, and IL-1β. These reductions in inflammatory markers were accompanied by reduction in amyloid plaque and size in the brains of 5xFAD mice compared to controls.

**Conclusion:**

Microglial amylin receptors mediate Aβ-evoked inflammation, and amylin receptor antagonists therefore offer an attractive therapeutic target for intervention in AD.

**Electronic supplementary material:**

The online version of this article (10.1186/s12974-017-0972-9) contains supplementary material, which is available to authorized users.

## Background

Alzheimer’s disease (AD), the most common form of dementia, is characterized by synaptic loss, deposition of misfolded proteins (amyloid beta protein, Aβ, and hyperphosphorylated tau), and neuroinflammation [1]. Microglia, the main immune cells in the brain, appear central to the initiation and progression of neuroinflammation in AD [2–4]. These cells are activated and recruited to amyloid plaques, phagocytose Aβ, and secrete cytokines [5]. Initiation of such an inflammatory cascade results from Aβ recruitment of intracellular cytosolic multiprotein complexes termed “inflammasomes” and the subsequent activation of microglial caspase-1 [6–8]. The inflammasome NLRP3, a member of the PYHIN family of proteins, and ASC, an adaptor protein, are deemed to be important in the context of neurodegenerative processes [9]. Caspase-1 generates inflammatory responses through cleavage and release of the injurious cytokines, IL-1β and IL-18, on to adjacent neurons [6, 10]. The release of such inflammatory cytokines also elicits upregulation of Aβ via increased APP processing [11], thus perpetuating a vicious cycle of Aβ-induced neuronal damage. Therefore, mechanisms whereby Αβ activates microglia to initiate this inflammatory cascade are crucial to understanding the interplay between microglia and neuronal viability in AD. Αβ is postulated to be endocytosed into murine microglia [7] or alternately interact with a variety of putative target receptors on the microglial membrane [12, 13]. In the course of examining the presence of amylin receptors on neurons, we made the surprising observation that amylin receptors are also present on microglia [14, 15]. We thus hypothesized that microglial amylin receptors may serve as a portal of expression of Aβ-induced inflammatory responses.

Amylin receptors (AMYs) consist of dimerized calcitonin receptor (CTR) with receptor activity-modifying proteins (RAMPs) and belong to class B GPCR. Amylin, the endogenous peptide that binds to the amylin receptor, mediates glycemic regulation, control of energy balance, and cognitive processes and modulates innate immunity through regulatory T cells [16–18]. We have previously shown that the deleterious effects of Aβ on cultured human and rat neurons are expressed through the amylin receptor and that amylin receptor antagonists, such as AC253, are neuroprotective against Aβ toxicity [14, 19]. Moreover, application of amylin receptor antagonists reverses impairment of Aβ or human amylin (hAmylin)-induced depression of hippocampal long-term potentiation, a cellular surrogate of memory [20]. Herein, we show that amylin receptors are not only present on human fetal and murine microglia but are functional in mediating Aβ-induced activation of an intracellular inflammatory cascade that results in the release of cytokines. Chronic systemic administration of cyclized form of the amylin receptor antagonist, AC253 (cAC253), which is readily brain penetrant, reduced markers of microglial activation and cytokine release in a mouse model of AD [15]. We also observed a parallel decrease in amyloid plaque formation in cAC253-treated AD mice.

## Methods

All experiments were conducted in compliance with the guidelines set by the Canadian Council for Animal Care and with the approval of the Human Research Ethics Board and Animal Care Use Committee (Biomedical Sciences) at the University of Alberta.

### Cell cultures

Two different types of microglial, primary cultures of human fetal microglia and mouse microglial BV2 cell line, were used for in vitro testing of amylin receptor function. This approach provided confirmation of the presence of microglial amylin receptors across species and cross-validated our observations on the function of amylin receptors in both primary cell cultures and a frequently used cell line. Primary mixed glial cultures were prepared from 12- to 15-gestational-week fetuses as previously reported [21, 22]. Briefly, the meninges and blood vessels were removed and the brain tissue was washed in minimum essential medium and mechanically dissociated by repeated trituration through a 20-gauge needle. Cells were centrifuged at 1500*g* for 10 min and re-suspended in minimum essential medium with 10% fetal bovine serum. The cultures were grown in a 5% CO_2_ humidified incubator at 37 °C. After mixed glial cultures were completely confluent, human fetal microglia (HFM) were isolated by shaking flasks at 100 rpm (IKA KS-260, IKA Works, Wilmington, NC) for 1 h at 37 °C. The media was then collected and centrifuged at 2500 RCF for 5 min at 4 °C. Cell pellets were re-suspended in microglia plating media (MEM + 10% FBS + 1% penicillin/streptomycin). HFM were plated at a 2 × 10^5^ cells per milliliter density, and microglia were allowed to attach overnight before further use for experiments. BV2 cells (kindly provided by Dr. T. Trang, University of Calgary) were cultured in DMEM/F12 media with 10% FBS. For all in vitro experiments, primary human fetal microglial cultures and BV2 cell cultures, each experiment was performed on a fresh batch of cell cultures and repeated a minimum of four times. Statistical analysis was performed on mean data.

In order to characterize the antagonist activity of cAC253 at subtypes of amylin receptors, we compared hAmylin-generated cAMP responses in stably expressed AMY1, AMY2, and AMY3 and CTR receptors in HEK293 cells and wild-type HEK (HEK-WT) cells (Additional file 1).

### Western blot

Frozen brain tissues or cultured cells were homogenized in cold RIPA buffer with protease inhibitors and proteins were quantified with BCA assay (BioRad, Mississauga, ON, Canada). Proteins were loaded at 50 μg per lane on a 12% polyacrylamide gel. Proteins were transferred to nitrocellulose membrane and then blocked with LiCor blocking buffer. Blots were further incubated with primary antibodies overnight at 4 °C on a shaker. Primary antibody used for Iba1 (1:500, rabbit, Wako), CD68 (1:500, mouse monoclonal, Dako), caspase-1 (1:1000, rabbit, Abcam), NLRP3 (1:1000, rabbit, Millipore), and β-actin (1:10,000 mouse, Sigma-Aldrich). IRDye 800CW goat anti-rabbit and IRDye 680CW goat anti-mouse (LiCor, 1:10,000) were used as secondary antibodies. Blots were imaged using LiCor Odyssey image system.

### ELISA (enzyme-linked immunosorbent assay)

Mouse brain cytokines, TNFα (Invitrogen), IL-6 (interleukin-6, Abcam), and IL-1β (Thermoscientific) were quantified using colorimetric ELISA kits following the protocol provided. In brief, hemi-brains were homogenized on ice for 3 h in RIPA buffer with protease inhibitor. The homogenized brain was centrifuged at 21,000*g* for 20 min at 4 °C. The supernatant was collected and diluted with PBS buffer pH 7.4 (1:100) prior its plate loading. Standard curves were plotted using the cytokine standards provided in the ELISA kits. All samples were analyzed in duplicate. The plate is measured at 450 nm and data expressed as pg/mg wet tissue.

### Cytokines PCR assay

The PCR array for TNFα, IL1β, and IL23 gene expression used the RT^2^ Profiler PCR Array system (96-well assay plate, SABiosciences) following product instruction. Briefly, HFM were plated in a six-well plate for 24 h, pre-treated with/without AC253 10 μM for 24 h, and then exposed to either hAmylin (1 μM) or Aβ_1–42_ (1 μM) for 6 h. RNA was isolated with Rneasy Mini kit (Qiagen) as per the product instructions. For each PCR Array, 4 μg of total RNA were used to prepare cDNA with the appropriate first strand kit from SABiosciences. The cDNA was characterized on the iCycler® iQ Real-Time PCR System (Bio-Rad Laboratories) using the RT2 Profiler PCR Arrays. The resulting raw data were then analyzed using the PCR Array Data Analysis Template and expressed as gene expression fold-change after treatment compared to the control untreated HFM cultures.

### Immunofluorescent-histological staining

For cultured cell immunofluorescent staining, cells were plated on Lab-TEK chamber slide (ThermoFisher) and fixed with 4% paraformaldehyde (PAF) in PBS, then permeabilized with 0.3% Triton X-100 in PBS and blocked with 2% BSA + 10% goat serum for 1 h. Primary antibodies used Iba-1 (1:100, Dako), CTR (1:50, ThermoFisher, Catalog # PA1-84457, Lot # QJ2098825 and RI2275863), RAMP3 (1:100, Abcam), Aβ (6E10, 1:300, Covance), and NLRP3 (1:300, Millipore) followed fluorescent secondary antibody (goat anti-mouse Alexa Fluor-546 and goat anti-rabbit Alexa Fluor-488) and were nuclear-stained with DAPI (0.1 μg/ml, ThermoFisher). Fluorescent dyes, DyLight-594 Lectin (Vector), HiLyte 555-labled Aβ_1–42_ (AnaSpec), and caspase-1 (FAM-FLICA caspase 1 kit, ImmunoChemistry Technologies) were used following product instruction. Fluorescent microscopy images were acquired with an Axioplan-2 fluorescence microscope with AxioVision software (Carl Zeiss Ltd., Toronto, ON, Canada).

For human brain immunohistochemical staining, 20-μm-thick brain sections were cut from 1-cm-thick coronal block of the human inferior temporal gyrus (IFG) of an AD brain (Braak Stage 6) obtained from the Netherlands Brain Bank. Prior to immunohistochemistry, the sections were pretreated with absolute methanol and 3% hydrogen peroxide for 10 min to avoid visualizing endogeneous peroxidase activity, followed by three 10 min (3 × 10 min) washes in TBS (Tris-buffered saline). Then, they were incubated overnight at 4 °C with the rabbit polyclonal anti-CTR antibody (ThermoFisher Scientific) at a working dilution 1:20 in Supermix (TBS containing 0.3% Triton X-100 and 2.5 mg/ml gelatin). The next day, the sections were washed in TBS (3 × 10 min) and then incubated in biotinylated goat anti-rabbit IgG, diluted 1:400 in Supermix for 2 h at room temperature. Following incubation, the sections were washed in TBS (3 × 10 min) and processed by the avidin-biotin complex (ABC) (Vector Laboratories, Burlingame, CA) for 2 h at room temperature. After washing in TBS (3 × 10 min), reaction products of dark blue color were visualized with 3,3′-diaminobenzidine-4HCl (20 mg/100 ml, Sigma) in TBS containing 0.2% ammonium nickel sulphate and 3% H_2_O_2_. Several sections were immunostained for CTR as mentioned above and after final washing in TBS were incubated overnight at 4 °C with either Iba-1 antibody (1:100, Dako) or RAMP3 rabbit polyclonal antibody (Abcam, Catalog # ab78017, Lot # 816327; Catalog # ab197372, Lot # GR2042843) at working dilution of 1:500 in Supermix. The next day, the sections were washed in TBS (3 × 10 min) and then incubated in biotinylated goat anti-rabbit IgG diluted 1:400 in Supermix for 2 h at room temperature. Further, the sections were washed 3 × 10 min in TBS and processed with ABC for 2 h at room temperature. After washing 3 × 10 min in TBS, the sections were treated with 3,3′-diaminobenzidine-4HCl (20 mg/100 ml, Sigma) in TBS containing 3% H_2_O_2_, resulting in the reaction product of yellow color. Thus, after CTR + Iba-1 dual immunostaining of the IFG sections, the neuronal final reaction product of dark blue color was associated with the presence of the CTR whereas deposit of yellow color was coupled with Iba-1 or RAMP3. Brightfield images were acquired on Axioplan 2 microscope (Zeiss) with AxioVision software (ver 4.8.2, Zeiss). The ability of the CTR and RAMP3 antibodies to detect these proteins was verified using AMY3-HEK293 transfected cells and wild-type HEK293 cells (Additional file 1 and Additional file 2: Figure S1A). We also performed siRNA RAMP3 transfections of BV2 microglial cells and compared detection of the RAMP3 protein in these cells compared to non-transfected cells (Additional file 1 and Additional file 2: Figure S1B).

For in-cell Western blot cyclic adenosine monophosphate (cAMP) quantification, mouse monoclonal anti-cAMP (R&D Systems) was used as a primary antibody and IRDye 700 goat anti mouse antibody (LI-COR) was used as a secondary antibody. Plates were imaged using an Odyssey Infrared Imaging System (LI-COR), and the integrated intensity was normalized to the total cell number on the same well as previously described [23].

### Ca^2+^ imaging

Ca^2+^ concentration were monitored with confocal microscopy as described previously [23, 24]. Briefly, HFMs were plated on glass coverslips pre-coated with poly-l-ornithine and incubated at 37 °C for 12–36 h. Then, incubated with 5 μM of membrane-permeant fluorescent Ca^2+^-sensitive dye Fluo-8L-AM (AAT Bioquest, Inc., Sunnyvale, CA) for 40 min at room temperature (20–23 °C) within < 2 h before imaging. For Ca^2+^ imaging, the superfusate of ion content similar to extracellular brain fluid thus contain the following: 130 mM NaCl, 4 mM KCl, 1 mM MgCl_2_, 2 mM CaCl_2_, 10 mM HEPES, and 10 mM glucose (pH 7.35) was applied at a rate of 3 ml/min using a roller pump (Watson-Marlow Alitea, Sin-Can, Calgary, AB, Canada). Aβ_1–42_ and hAmylin were dissolved in sterile bidistilled water at 1 mM stock solution and incubated at room temperature for 10 min before dilution with superfusate for the final used concentrations. Fluorescence intensity was monitored with a FV-300 laser-scanning confocal microscope (Olympus FV300, Markham, Ontario, Canada) equipped with an argon laser (488 nm) and excitation/emission filters (FF02-520/28-25; Semrock, Inc.) for an emission wavelength at 514 nm, measured with a numerical aperture of 0.95 20× XLUMPlanF1 objective (Olympus). Images were acquired at scan rates of 1.25–1.43 per second using a 2–3× digital zoom at full-frame (512 × 512 pixel) resolution. Regions of interest were drawn around distinct cell bodies, and analysis of time courses of change in fluorescence intensity were generated with FluoView software (version 4.3; Olympus).

### Peptides and reagents

AC253 peptide was cyclized to improve its stability and brain penetration when administered systemically [15]. To cyclize the AC253 peptide (cAC253) via the flanking d-cysteines, the crude peptide (61.2 mg) was dissolved in 0.1 mM Tris buffer pH 8.3 having 20% of DMSO to accelerate disulfide bond formation, and the mixture was stirred at room temperature in an open flask for 48 h and further purified on RP-HPLC (reversed phase high-performance liquid chromatography).

Soluble oligomeric Aβ_1–42_ or the inverse sequence peptide Aβ_42–1_, hAmylin, and AC253 were prepared according to published protocols [14, 25]. Human amylin and AC253 were purchased from American Peptide (Sunnyvale, CA) and Aβ peptides from rPeptide (Bogart, GA). The human amylin was prepared in 1 mM stock solution in water and further diluted to the final application concentration with cell culture media as previously described [14].

### In vivo experiments and mouse brain tissue processing

For in vivo experiments, intraperitoneal injection (ip) administration of cAC253 was carried out in transgenic 5XFAD mice [15], and wild-type littermate control mice (both male and female) were obtained from Dr. David Westaway (University of Alberta). These mice were equally and randomly distributed into four groups, Tg-NS (*n* = 10), Tg-cAC253 (*n* = 10), Wt-NS (*n* = 10), and Wt-cAC253 (*n* = 10). Mice received ip either normal saline (NS) or cAC253 (200 μg/kg) three times a week starting at 6.5 months of age for 5 weeks. Mice were housed under standard laboratory conditions (12/12-h light/dark cycle, lights on at 0600 h) with a room temperature of 21 °C. Water and food were available ad libitum. After completion of treatments, all mice were sacrificed with an overdose of isoflourane anesthetic and perfused transcardially with saline, and the brains were harvested. The right hemisphere was frozen for biochemical analysis (Western blot, ELISA), and the left hemisphere was fixed with PAF for 4 h at 4 °C. These brain tissues were further processed with modified CLARITY protocol (http://www.chunglabresources.com/cl1#cl-protocol). Briefly, the fixed brain tissue was transferred to hydrogel monomer solution (4% acrylamide, 4% PFA, and 1xPBS (phosphate-buffered saline)) at 4 °C for 24 h and, subsequently, to a 24-well plate, merged in fresh hydrogel solution, and the tissue was brought to 37 °C till formation of the gel. Thick sagittal slices (400 μm) were cut on an HR2 Slicer (Sigman Electronic, Germany). The thicker brain sections were cleared with 8% SDS in PBS for 24 h, followed by 0.3% Triton X-100 in PBS for 24 h, blocking the section in 2%BSA-10% goat serum for 4 h. A modified thioflavin S staining was used for detecting Aβ plaques. Briefly, the brain sections were rinsed with distilled water, dropped with thioflavin S (0.0125% in 50% ethanol) for 5 min, further washing with 50% ethanol and water. Then, these sections were incubated with CD68 antibody (1:50, DAKO) at 4 °C for overnight. After washing with PBS, these sections were incubated with goat-anti mouse Alexa-fluor546 (1:200, Invitrogen) at RT for 4 h. Sections were cleared with 8% SDS for 24 h followed by a 24-h wash with PBS-Triton-X100 solution. Images were visualized using fluorescence microscopy (Axioplan-2, Carl Zeiss Ltd). Amyloid plaque size and area were analyzed with Image J software.

### Statistical analysis

The statistical data are presented as mean ± SEM unless otherwise specified. Significance was determined by one-way analysis of variance (ANOVA), followed by Tukey’s post hoc test with Prism software (GraphPad Prism 5, GraphPad Software, San Diego, CA). Differences between groups were considered to be significant at *p* < 0.05.

## Results

### Microglial cells expressed functional AMY receptors

**Fig. 1:**
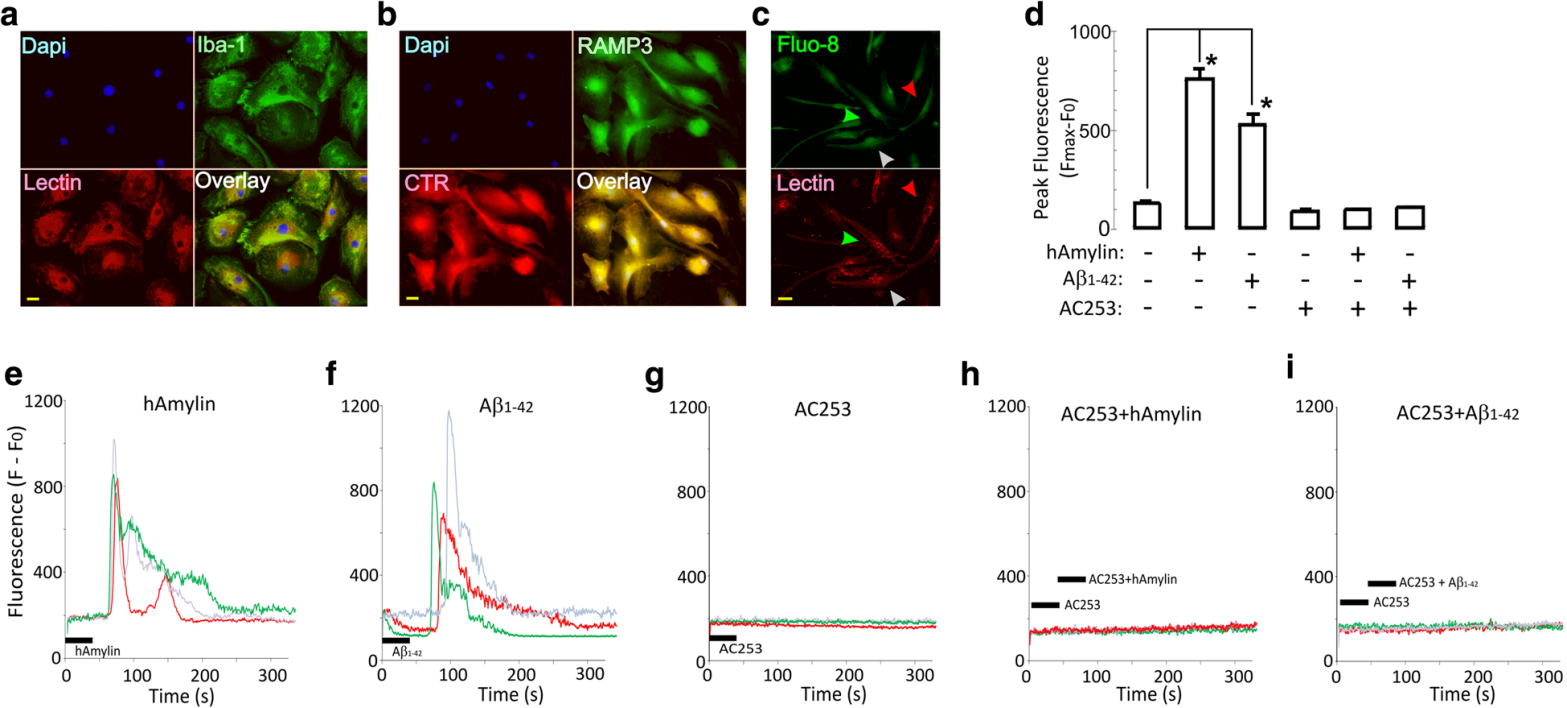
Microglial cells express functional amylin receptors. **a** Primary cultures of human fetal microglia (HFMs) that are stained with microglial antibody, Iba-1 (green), and DyLight-594-labeled lectin (red). **b** These primary cultured HFMs were also stained for the two dimeric proteins that are components of the amylin receptor 3 (AMY3) subtype, the calcitonin receptor (CTR), and the receptor-associated membrane protein 3 (RAMP3). **c** Cultured HFMs were loaded with the fluorescent intracellular calcium dye, Fluo-8L-AM (green), and with lectin (red), an in vivo microglial marker. Arrowheads indicate cells from which Ca^2+^ signals were recorded. The same cell culture is also stained with lectin (red). The field in **c** shows that a majority of cells are microglia. **d** Summary of data on intracellular calcium changes after human amylin (hAmylin) and Aβ_1–42_ without and with application of the amylin receptor antagonist, AC253 in HFM (*p < 0.05, *n* = 123 cells in 12 culture wells from four different batch of the culture cells). The candidate traces for intracellular calcium changes are illustrated in **e**–**i**. Elevations of Ca^2+^induced by acute (30 s) application of either hAmylin (1 μM, **e**) or Aβ_1–42_ (1 μM, **f**). The changes in Ca^2+^ were blocked by AC253 (10 μM, **g**–**i**). Traces correspond to cells identified in **c** with arrowheads. Scale bar = 20 μm

**Fig. 2:**
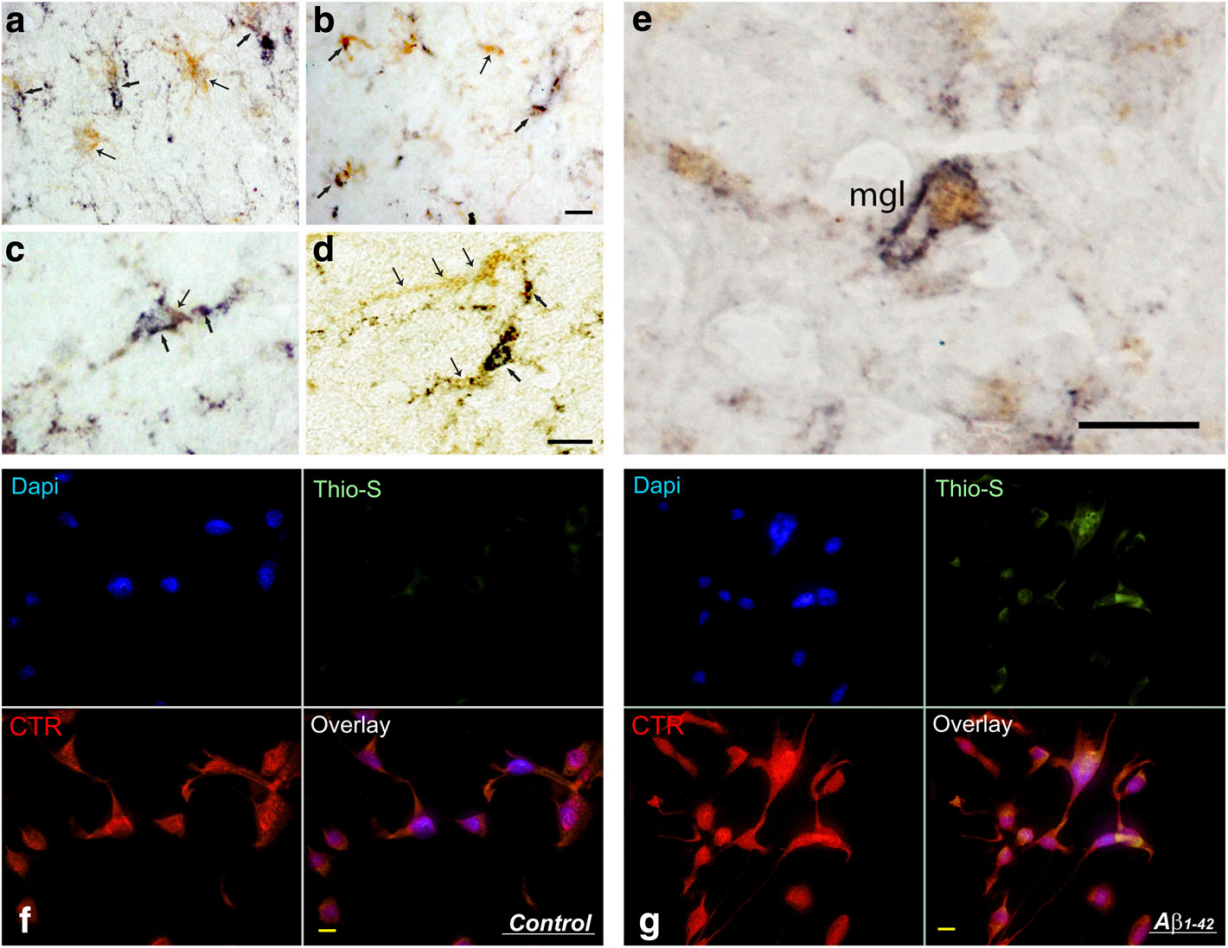
Amylin receptors on microglia in human Alzheimer’s disease brain and BV2 microglial cells. **a**, **b** Section from the inferior frontal gyrus of the AD patient (Braak Stage 6) after dual immunostaining both with the antibody to microglial marker Iba-1 (yellow) and calcitonin receptor (CTR) (dark blue). Arrows show microglial cells expressing both Iba-1 and CTR; thin arrows point to microglial cells expressing only Iba-1. **c**, **d** Single microglial cells are presented at a higher magnification, with thin arrows pointing to Iba-1 location (yellow) and arrows pointing to the CTR location (dark blue). **e** Coronal section from the inferior frontal gyrus of the AD patient after double immunohistochemical staining with antibody against CTR and receptor activity modifying protein 3 (RAMP3). Notice the microglia-like profile (mgl) expressing both CTR (blue) and RAMP3 (yellow). **f** BV2 microglial cells expressed CTR (red). **g** These BV2 cells were cultured in the presence of Aβ_1–42_ 1 μM for 24 h. The Aβ was taken up into the cells and formed plaque-like structures stained with thioflavin S (green). Scale bars = 10 μm

In primary HFM cultures, the microglia population comprises more than 90% of cells as judged by staining with microglial markers, Iba-1, and DyLight 594 lectin (Fig. 1a). To establish AMY expression in these microglial cells, we first immunohistochemically identified the presence of CTR and RAMP3 (Fig. 1b). We next assessed functionality of AMYs using live-cell calcium imaging (with Fluo-8) and confocal microscopy of identified microglia (Fig. 1c). Bath applications of either monomeric human amylin (hAmylin) or soluble oligomeric Aβ_1–42_ (1 μM) to HFM for 30 s produced a robust Ca^2+^ increase within 1 min after entry of the peptide within the imaging chamber. These Ca^2+^ increases displayed a sharp peak and returned to base line within 2 min (Fig. 1e, f), and they are similar to responses evoked by these peptides in HEK293 cells expressing the AMY3 receptor subtype [23]. The transient elevations in Ca^2+^ due to hAmylin or Aβ_1–42_ applications were abolished by prior application of the amylin receptor antagonist, AC253 (Fig. 1h, i), suggesting that these responses are AMY receptor-mediated. AC253 applied alone did not evoke any alterations in intracellular Ca^2+^ levels (Fig. 1g). Pooled data shown in Fig. 1d are from 123 HFM in 12 culture wells from four different batches of HFM cultures. Finally, we also confirmed, using inferior temporal gyrus sections from autopsied AD patient brain, that adult human microglia were immunopositive for CTR and RAMP3 (Fig. 2a–e). In addition, BV2 cells, a mouse microglia cell line, also expressed amylin receptors (Fig. 2f, g). In these cells, exposure to Aβ_1–42_, (1 μM) resulted in an uptake of the peptide into the microglia and formation of plaque-like structures on the cell surface as determined by thioflavin S staining (Fig. 2g).

### Aβ induces AMY receptor-mediated microglial immune responses

**Fig. 3:**
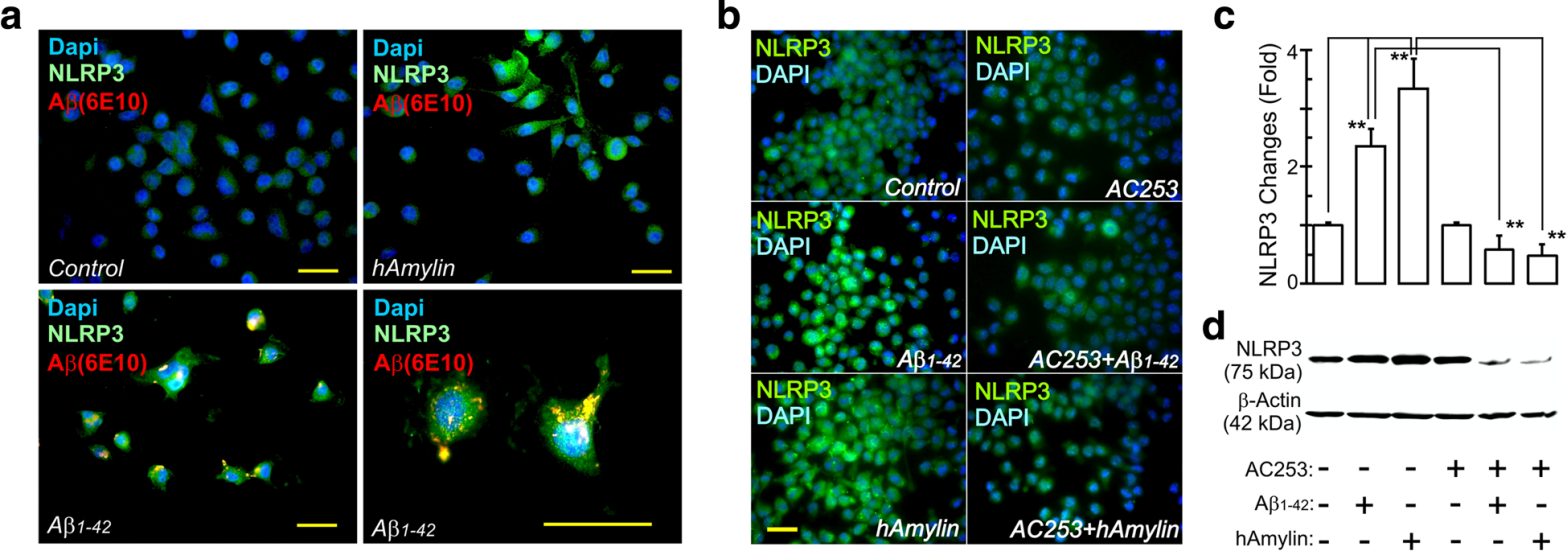
Amylin receptor mediates activation of inflammasomes in microglia. **a** Increased activation of the inflammasome, NLRP3 (green), following application of either Aβ_1–42_ or hAmylin human fetal microglial (HFMs) cultures. Aβ is identified with immunostaining with 6E10 antibody and shows as yellow on account of its co-localization with NLRP3. **b** BV2 cells (murine microglial cell line) treated either Aβ_1–42_ or hAmylin (2 μM) for 24 h show a similar increase in NLRP3 staining as in HFMs that is attenuated by 16 h pre-treatment with AC253 (10 μM). **c** Summary of amylin receptor mediated changes in NLRP3 observed and quantitated from Western blots, an example of which is shown in **d**. **p < 0.01. Scale bar = 20 μm

We next sought to examine the potential role of microglial AMY receptors in Aβ-induced activation of the intracellular inflammasome cascade. We observed levels of NLRP3, the best studied of inflammasomes in the context of AD, to be low under basal conditions but increased significantly after hAmylin or Aβ stimulation (Fig. 3a). This stimulated increase in the NLRP3 expression was attenuated in the presence of the AMY antagonist, AC253, thus identifying a role for AMY receptors in activation of the inflammasome cascade (Fig. 3b). Western blot analysis further confirmed AMY receptor-mediated changes in NLRP3 expression (Fig. 3c, d).

**Fig. 4:**
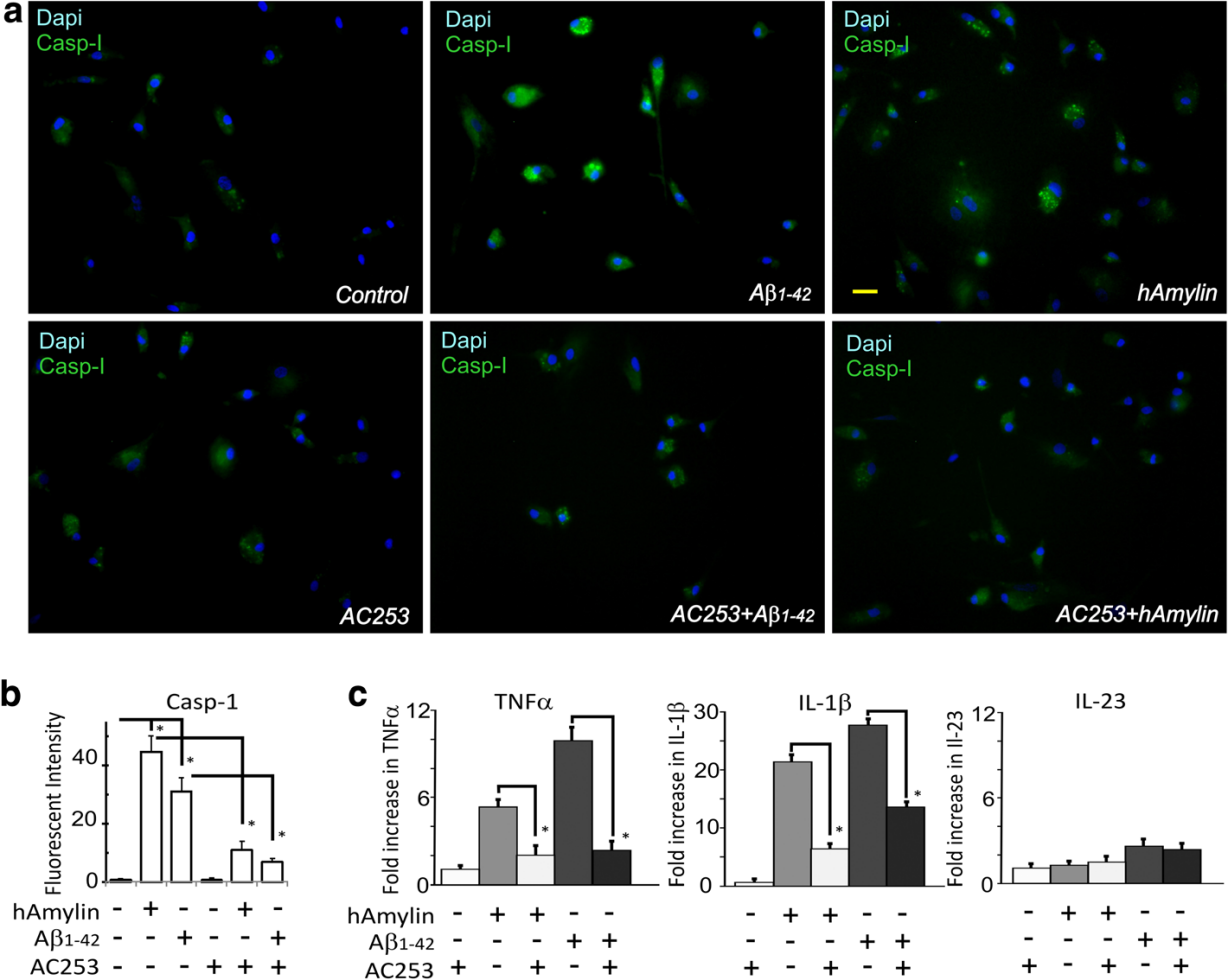
Aβ induces upregulation of caspase-1 and pro-inflammatory cytokines in human fetal microglia (HFMs) via amylin receptor. **a**, **b** Aβ_1–42_ or hAmylin increase caspase-1 expression that is detected by a fluorometric assay (green). Pre-treatment of HFMs with amylin receptor antagonist, AC253 blocks caspase-1 expression. **c** RT-PCR detection of cytokines (TNFα, IL-10, IL-1β, and IL-23) in HFMs treated with either hAmylin, Aβ_1–42_, or AC253. Pre-treatment of HFMs with AC253 markedly attenuates upregulation of TNFα, IL-10, and IL-1β, but not IL-23. *p < 0.01. Scale bar = 20 μm

Since inflammasomes function as intracellular sensors for danger signals and in response trigger activation of caspase-1 and subsequent cleavage and release of cytokines, we also examined the role of AMY receptors in these downstream events. Both Aβ and hAmylin increased caspase-1 expression in HFM and BV2 cell cultures, a response that was blunted by AC253 (Fig. 4a, b). Additionally, using RT-PCR assay, we also identified Aβ and hAmylin-mediated increases in IL1β, and TNFα, but not IL23, that were significantly attenuated by AC253 (Fig. 4c).

### In vivo therapeutic targeting to block AMYs reduces brain inflammation and amyloid plaque formation in transgenic animal model of AD

**Fig. 5:**
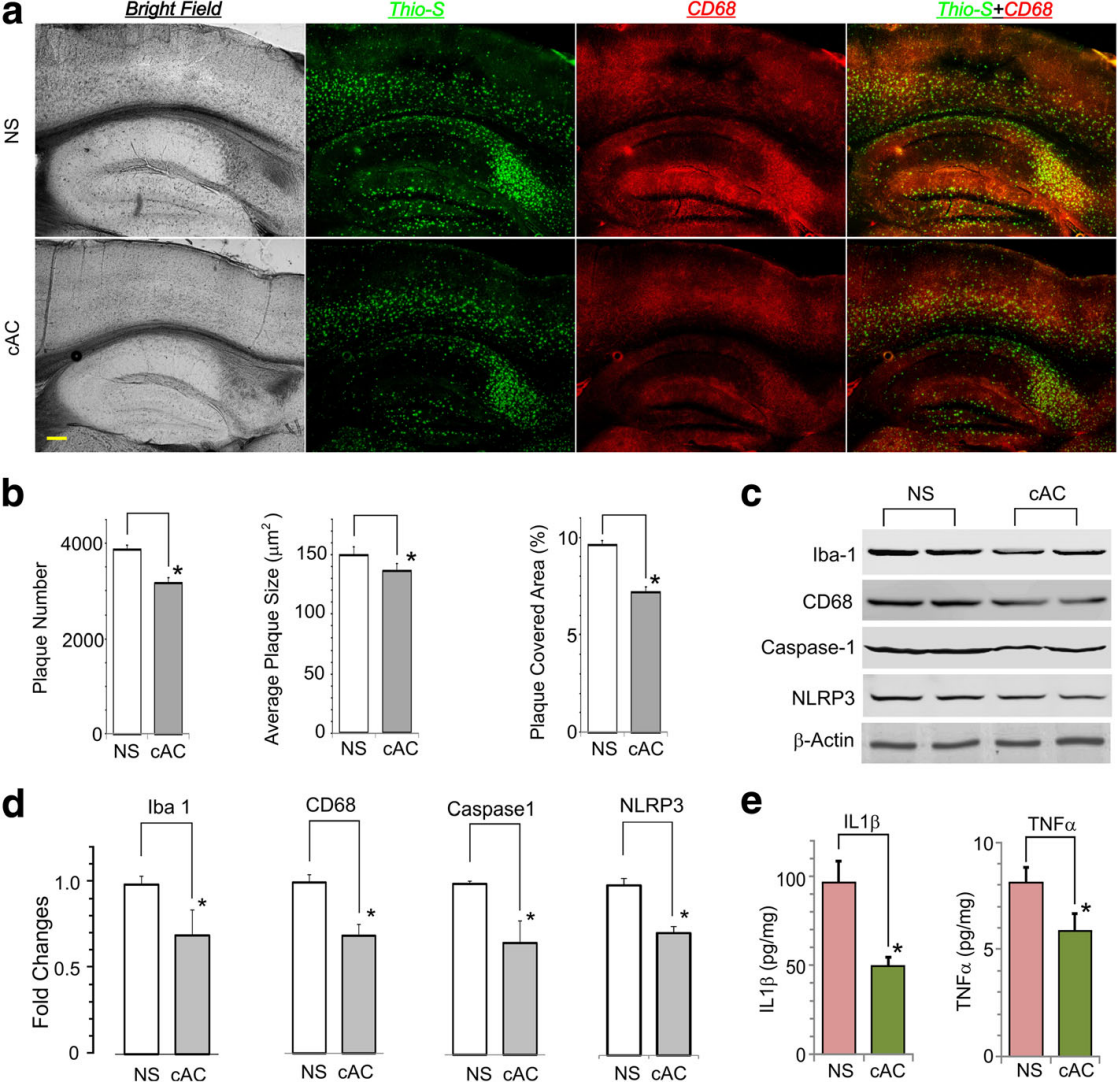
Blocking the amylin receptor reduces amyloid plaque formation and inflammatory markers in transgenic AD mice. **a** Transgenic AD mice (5xFAD), amyloid plaques in brains of treated with cyclized AC253 (cAC) or normal saline (NS) mice reveals significant reduction of the amyloid plaque (green, stained with thioflavine S). The activated microglia (red, stained with CD68 antibody) are also reduced. The total amyloid plaque number and plaque size are significantly reduced (**b**). **c** Markers of brain microglial activation (Iba-1, CD68) and inflammation (caspase-1 and TNFα) in the two groups of 5xFAD mice (NS, cAC253) were detected by Western blot and quantified (**d**). **e** ELISA analysis of brains from the same animals show**s** that IL-1β and TNFα are significantly reduced with cAC253 treatment. *p < 0.05. Scale bar in **a** = 200 μm

We have recently synthesized a cyclized form of AC253 (cAC253), which retains the neuroprotective properties of the linear form of the peptide but is proteolytically stable and highly brain-penetrant when administered intraperitoneally [15]. The pKa = 6.942 for the cAC253 peptide was calculated from Molecular Operating Environment (MOE) from Chemical Computing Group (Montreal, QC, Canada). The cAC253 also demonstrated a higher level of antagonist activity at AMY receptors with IC50, 0.3 μM, versus AC253, 0.85 μM, using in-cell western blot detection [15]. In HEK293 transfected cells, cAC253 demonstrated similar activity as AC253 and blocked hAmylin activation of AMY3 and AMY1 receptors (Additional file 3: Figure S2). We also tested the ability of cAC253 (at different concentrations) to competitively antagonize human amylin-generated cAMP responses across full range of concentrations. We examined the effects of chronic systemic administration of cAC253 on changes in amyloid pathology and inflammatory markers in a transgenic mouse model of AD, 5xFAD. Six and a half-month-old 5xFAD mice that received 5 weeks of cAC253 treatment (ip, three times a week) demonstrated significant reduced amyloid plaque formation, and we observed less number of activated microglial cells compared to 5xFAD mice treated with normal saline (NS) injections (Fig. 5a, b). Aβ deposition was significantly reduced as measured by either the number of Aβ plaques, or total area of Aβ-positive profiles in the cAC253-treated 5xFAD animals compared to those receiving NS (Fig. 5b). Wt mice showed no Aβ deposits. Protein expression levels of microglial markers Iba-1 and CD68 (activated microglia), inflammasome NLRP3, and caspase-1 were significantly reduced (approximately 30%) in cAC253-treated 5xFAD group compared to NS treatment (Fig. 5c, d). Additionally, levels of cytokines IL-1β and TNFα measured by ELISA were also significantly reduced after ip cAC253 treatment (Fig. 5e).

## Discussion

Although recent studies indicate that amylin and amylin receptor are involved in microglia-mediated neuroinflammation in AD mice [26, 27], this study shows for the first time immunohistochemical presence of amylin receptors on human and murine microglia, which serve as a portal for the expression of the inflammatory effects of Aβ in the brain. Specifically, amylin receptors present on human microglia are functional in that applications of either monomeric hAmylin or soluble oligomeric Aβ_1–42_ results in an increase in cytosolic Ca^2+^, a response that is accompanied by activation of the inflammasome, NLRP3, and caspase-1 with the subsequent release of pro-inflammatory cytokines, IL-1β and TNFα. Blockade of the amylin receptors with an amylin receptor antagonist, AC253, results in an attenuation of these inflammatory mediators. In a transgenic mouse model of AD, systemic administration of cAC253 leads to an improvement in spatial memory and learning [15], that is, accompanied by diminution of the amyloid burden, and an attenuation of microglial markers within the brain. Collectively, these observations suggest a possible role for the amylin receptor as a target in the therapeutics of AD.

**Fig. 6:**
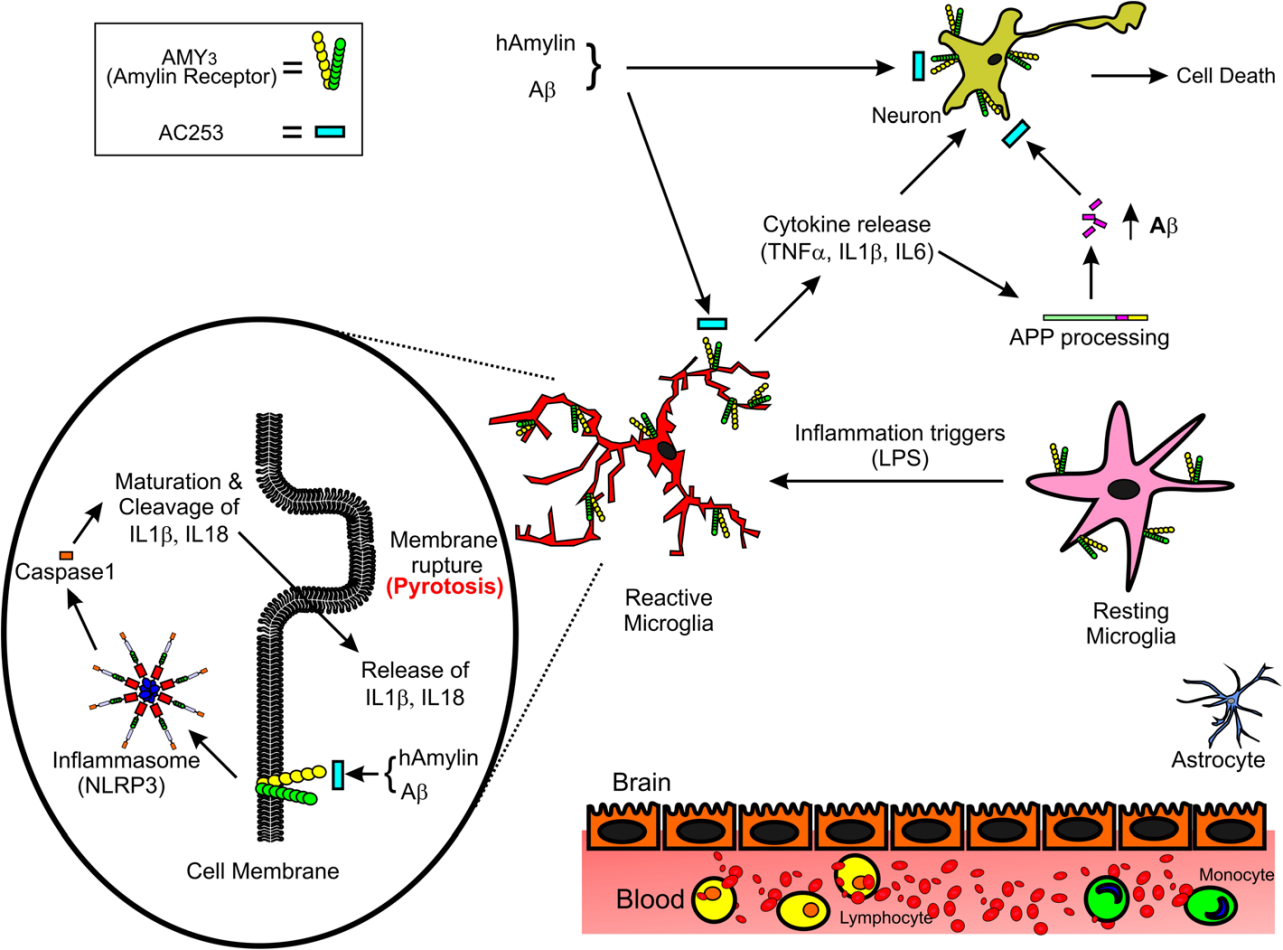
Proposed model for the role of microglial and neuronal amylin receptors in mediating the amyloid beta (Aβ)-induced neurodegeneration. Amylin receptors, comprised of calcitonin receptor (CTR) and receptor activity modifying protein 3 (RAMP3), are expressed on both neurons and the microglia. The expression of amylin receptors on microglial cells is increased in response to inflammatory triggers, such as lipopolysaccharide (LPS), resulting in activation of these cells. Interaction of Aβ with amylin receptors on the activated microglia leads to increased production and release of cytokines, which act directly on neurons to produce cell death and additionally augment the production of Aβ via processing of the amyloid precursor protein (APP). This Aβ, in turn, interacts with neuronal and microglial amylin receptors to produce cell death. The amylin receptor antagonist AC253 acts on *both* the neuronal and microglial receptors to block the detrimental effects of Aβ and affords protection against the neuronal degeneration

Increasing evidence suggests that AD pathogenesis involves multiple interacting compartments that include neuronal and synaptic disruption, vascular pathology, and neuroinflammation [1, 28]. Of these, neuroinflammation mediated primarily by microglia, which are critical regulators of immune responses, is a key feature of early AD. Microglia are implicated in clearance of Aβ either by phagocytosis or binding to receptors on microglial plasma membrane [28–33]. In vitro and in vivo studies have shown that consequences of Aβ interactions with microglia include lysosomal damage, activation of the NLRP3 inflammasome complex, and the release of cytokine IL-1β in a caspase-1-dependent manner [9]. The uncontrolled release of inflammatory cytokines also elicits upregulation of Aβ via increased APP processing [11], thus perpetuating a vicious cycle of Aβ-induced neuronal damage that is a feature of late AD [28]. Microglial receptors therefore present an attractive target to mitigate both neuroinflammation and neuronal toxicity induced by amyloid as depicted in our working model (Fig. 6). We have previously shown that a pharmacological blockade or genetic depletion of the amylin receptor in the human neuronal cell culture model protects against the Aβ-induced toxicity [14, 34]. Here, we show that blockade of microglial amylin receptors with an amylin receptor antagonist, AC253 in vitro, results in attenuation of NLRP3-mediated inflammatory cascade and diminished cytokine release. Furthermore, in a mouse AD model with systemic administration of cAC253 results in attenuation of key indices of brain inflammation-activated and reactive microglia (Iba-1, CD68), the inflammasome NLRP3, caspase-1, and pro-inflammatory cytokines, IL-1β and TNFα.

In transgenic mouse models of AD, microglial activation is observed to parallel accumulation of amyloid [35, 36] and a similar relationship has also been reported for the human AD using PET ligands [36, 37]. In our study, we administered the amylin receptor antagonist cAC253 intraperitoneally to 5xFAD mice at six and a half months of age, a time point corresponding to the presence of pathological features such as the amyloid accumulation and neuroinflammatory changes [15]. In these in vivo experiments, we chose to use cAC253 on account of its superior stability, pharmacokinetic profile, and brain penetrability when administered systemically compared with linear form of the peptide AC253 [15]. Five weeks post-treatment with cAC253, we observed a significant reduction in both the amyloid burden and indices of neuroinflammation in a manner similar to that observed in vitro. Based on these findings, we conclude that the amylin receptor blockade has the potential to mitigate Aβ-induced microglial activation, a key event in AD pathogenesis, and subsequent cytokine-induced neuronal death.

As a neuroendocrine hormone, amylin mediates glycemic regulation, energy balance control, and cognitive/motivational processes [18]. Human amylin also modulates autoimmunity and innate inflammation through regulatory T cells [16, 17]. Amyloid precursor protein (APP) and Aβ interact with variety of G-protein-coupled receptors (GPCRs) including amylin receptors [38]. Amylin receptors (AMYs) are a dimerized calcitonin receptor (CTR) with receptor activity-modifying proteins (RAMPs) and belong to class B GPCRs. We have previously shown that hAmylin and Aβ preferentially activate AMY3 receptor subtype resulting in downstream activation of MAPK and Akt signaling pathways that could be blocked with amylin receptor antagonist, AC253 [23]. At a functional level, application of either AC253 or pramlintide (marketed as a synthetic analog of amylin) attenuate hAmylin- and Aβ-induced depression of hippocampal long-term potentiation, a cellular surrogate of memory [20, 39]. Pramlintide when administered systemically to transgenic AD mice has been shown to reduce amyloid burden in the brain through promoting an efflux of Aβ across the blood brain barrier [40, 41]. Cyclized AC253 may reduce brain amyloid through a similar mechanism. Additionally, we suggest that cAC253, which readily crosses the blood brain barrier, could block both the neuronal and microglial amylin receptors, thereby diminishing the deleterious effects of elevated levels of Aβ in transgenic AD mice. As a consequence, cAC253-treated AD mice demonstrate not only improved spatial memory as we have reported [24], but also an attenuation of neuroinflammation.

## Conclusions

In this study, we provide evidence that functional amylin receptors are expressed on both human and murine microglia and mediate Aβ-induced activation of the inflammatory cascade of NLRP3 and caspase-1 with subsequent release of cytokines. Amylin receptor antagonism can attenuate these pro-inflammatory events in vitro. Chronic administration of such an antagonist results in improvement in amyloid burden and inflammatory markers in an AD mouse model. This supports the idea inflammatory changes in the brain that contribute to cognitive dysfunction in AD may, in part, be mediated via the amylin receptor. Furthermore, amylin receptor antagonists are capable of attenuating Aβ-evoked inflammation. Thus, amylin microglial receptors could provide novel treatment for AD specifically by targeting neuroinflammation, an early event in this disease.

## Additional files

Additional file 1: Supplemental material and methods. (DOCX 13 kb)

Additional file 2: Figure S1. A, Western blot showing AMY3 transfected HEK293 cells demonstrate a marked increase in level of expression of CTR and RAMP3 proteins compared to wild-type (WT) HEK cells. B, in BV2 cells, RAMP3 protein expression shows a marked decreased after RAMP3 siRNA transfection compared to the control non-transfected cells. (JPEG 1495 kb)

Additional file 3: Figure S2. Cyclic-AC253 (cAC) competitively blocks human amylin effects in a manner similar to AC253. A, Representative images (from in-cell western blots) for cAMP changes in AMY1–3-expressing HEK293 cells following exposure to hAmylin in the presence of increasing concentrations of cAC253. B and C, cAC253 blocked hAmylin-induced cAMP increases in a dose-dependent manner in AMY3- and AMY1-expressing HEK cells. The hAmylin activated AMY3 and AMY1 receptors but not significantly AMY2, CTR, and HEK wild-type control cells as previously observed (Fu et al., J. Biol. Chem. 2012). D, cAC253 blocked hAmylin responses in a dose-dependent manner in AMY3-HEK cells. *p < 0.05. (JPEG 635 kb)

## Abbreviations

ABC: Avidin-biotin complex
AD: Alzheimer’s disease
AMYs: Amylin receptors
ANOVA: One-way analysis of variance
Aβ: Beta-amyloid
cAC253: Cyclized form of the amylin receptor antagonist AC253
CTR: Calcitonin receptor
ELISA: Enzyme-linked immunosorbent assay
hAmylin: Human amylin
HFM: Human fetal microglia
LTP: Long-term potentiation
IFG: Human inferior temporal gyrus
ip: Intraperitoneal injection
NS: Normal saline
PAF: Paraformaldehyde
PBS: Phosphate-buffered saline
RAMPs: Receptor activity-modifying proteins
RP-HPLC: Reversed phase high-performance liquid chromatography
TBS: Tris-buffered saline
Tg: Transgenic
