# Supplementary material for: Role of microglial amylin receptors in mediating beta amyloid (Aβ)-induced inflammation
Source: J Neuroinflammation. 2017 Oct 6;14:199. doi: 10.1186/s12974-017-0972-9 (PMC5639602; doi:10.1186/s12974-017-0972-9)
Supplement: Supplementary file 1 — Supplemental material and methods. (DOCX 13 kb) [file 12974_2017_972_MOESM1_ESM.docx]

**Supplemental Material and Methods**

Amylin receptor subtypes stably expressed in human embryonic kidney (HEK293) cell-line were generated and characterized as described in a previous published article from our laboratory ( Fu et al., J. Biol. Chem. 2012). We used these same cell lines to further confirm the specificity of commercially purchased antibodies for CTR and RAMP3 (see below for details) and to characterize antagonistic effects of cAC253 at amylin receptor subtypes.

Western Blotting

Cultured cells (AMY3-HEK, WT-HEK293, and BV2 cells) were homogenized in cold RIPA buffer with protease inhibitors and proteins were quantified with BCA assay (BioRad, Mississauga, ON, Canada). Proteins were loaded at 50 μg per lane on a 12% (for CTR detection) and 16% (for RAMP3 detection) polyacrylamide gel. Proteins were transferred to nitrocellulose membrane, and then blocked with LiCor blocking buffer. Blots were further incubated with primary antibodies overnight at 4 °C on a shaker. Primary antibody used for CTR (1:500, rabbit polyclonal, ThermoFisher scientific), RAMP3 (1:500, rabbit polyclonal, Abcam), and β-actin (1:10000 mouse, Sigma-Aldrich). IRDye 800CW goat anti-rabbit and IRDye 680CW goat anti-mouse (LiCor, 1:10000) were used as secondary antibodies. Blots were imaged using LiCor Odyssey image system.

RAMP3 siRNA knock down

siRNA corresponding to the mouse RAMP3 genes, purchased from ThermoFisher Scientific (Silencer Pre-Designed siRNA, Catalog # AM16708). The lipofectamine RNAiMax reagent (ThermoFisher Scientific, catalog # 13778-150) was used for siRNA transfection followed the company protocol. Briefly, BV2 cells were plated at a density of 0.5x10^6^ cells/well in a 6-well plate and cultured for 24 hours before transfection. On the day of transfection, dilute RNAiMax reagent and RAMP3 siRNA with OptiMEM (ThermoFisher Scientific). The RAMP3 siRNA was next mixed with Lipofectamine RNAiMax and incubated for 5 minutes, and then added to the BV2 cell cultures. The 25 pmol of RAMP3 siRNA was used for each transfected well. Cultures were gently mixed and placed in an incubator at 37°C with 5% CO_2_ for 48 hours. These cells were lysed in cold RIPA buffer for further immunoblotting.

Quantitative cAMP Measurements

The in-cell Western blot cyclic adenosinemonophosphate (cAMP) quantification was used as previously described (Fu et al., J Biol Chem. 2012). Briefly, amylin receptor stable cells were plated at a density of 4x10^4^ cells/well in a 96-well plate and cultured for 24 hours. On the day of experiment, culture media was replaced with serum free DMEM medium and further incubate for 6 h before treatment. The cAMP was measured at 5 min after treatment, i.e., at 5 min after different concentration of hAmylin and/or cAC253 treatment, cells were fixed with 4% paraformaldehyde in PBS for 10 min. These fixed cells were permeabilized with PBS + 0.02% Triton X-100, 3x wash and 5 min each, and further blocked with LiCor blocking buffer for 2 h at RT. Followed with cAMP detection. The mouse monoclonal anti-cAMP (R&D Systems) was used as a primary antibody, and IRDye 700 goat anti mouse antibody (LI-COR) was used as a secondary antibody. Plates were imaged using an Odyssey Infrared Imaging System (LI-COR), and the integrated intensity was measured. Data were plotted, and nonlinear regression was fitted using Prism software (version 5, GraphPad Software, La Jolla, CA).
