# Supplementary figures and images for: Role of microglial amylin receptors in mediating beta amyloid (Aβ)-induced inflammation
Source: J Neuroinflammation. 2017 Oct 6;14:199. doi: 10.1186/s12974-017-0972-9 (PMC5639602; doi:10.1186/s12974-017-0972-9)

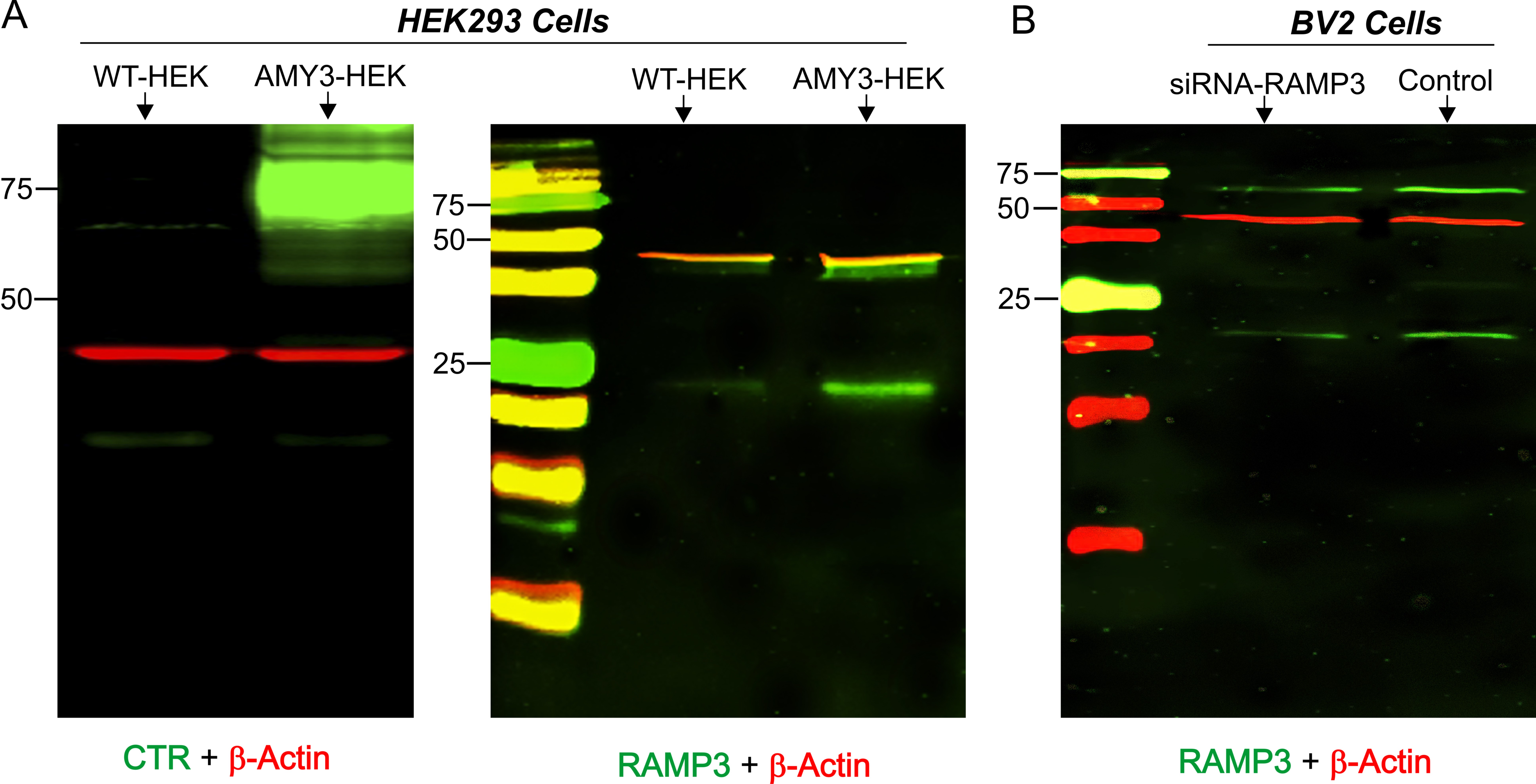

Supplement: Supplementary file 2 — A, Western blot showing AMY3 transfected HEK293 cells demonstrate a marked increase in level of expression of CTR and RAMP3 proteins compared to wild-type (WT) HEK cells. B, in BV2 cells, RAMP3 protein expression shows a marked decreased after RAMP3 siRNA transfection compared to the control non-transfected cells. (JPEG 1495 kb) [file 12974_2017_972_MOESM2_ESM.jpg]

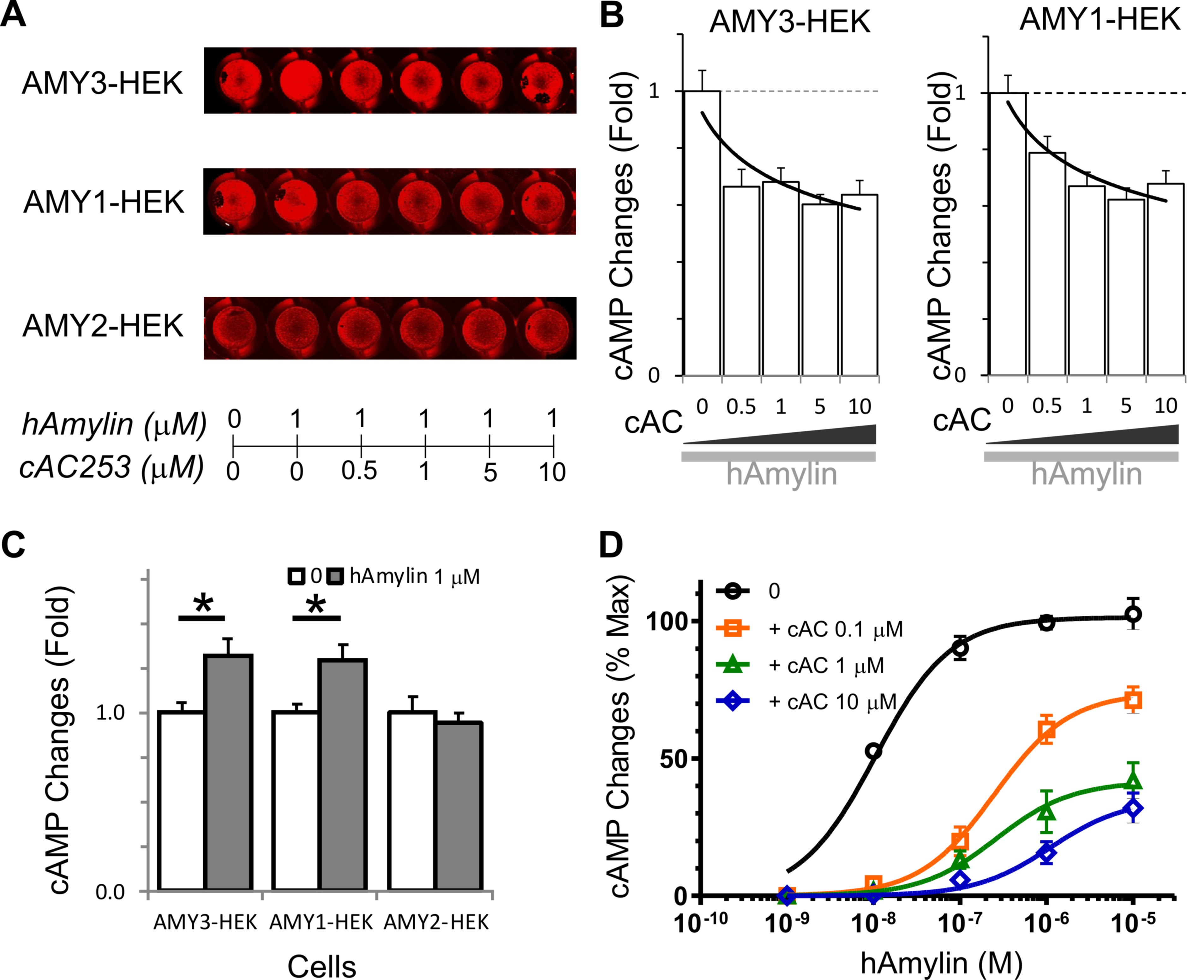

Supplement: Supplementary file 3 — Cyclic-AC253 (cAC) competitively blocks human amylin effects in a manner similar to AC253. A, Representative images (from in-cell western blots) for cAMP changes in AMY1–3-expressing HEK293 cells following exposure to hAmylin in the presence of increasing concentrations of cAC253. B and C, cAC253 blocked hAmylin-induced cAMP increases in a dose-dependent manner in AMY3- and AMY1-expressing HEK cells. The hAmylin activated AMY3 and AMY1 receptors but not significantly AMY2, CTR, and HEK wild-type control cells as previously observed (Fu et al., J. Biol. Chem. 2012). D, cAC253 blocked hAmylin responses in a dose-dependent manner in AMY3-HEK cells. *p < 0.05. (JPEG 635 kb) [file 12974_2017_972_MOESM3_ESM.jpg]
